# CCL2/C–C chemokine receptor type 2‐mediated interactions among mast cells, basophils, and endothelial cells

**DOI:** 10.1002/clt2.70044

**Published:** 2025-02-23

**Authors:** Maruša Rihar, Rajia Bahri, Vida Forstnerič, Silvia Bulfone‐Paus, Peter Korošec

**Affiliations:** ^1^ University Clinic of Respiratory and Allergic Diseases Golnik Golnik Slovenia; ^2^ Biotechnical Faculty University of Ljubljana Ljubljana Slovenia; ^3^ Lydia Becker Institute of Immunology and Inflammation Division of Musculoskeletal and Dermatological Sciences School of Biological Sciences University of Manchester Manchester UK; ^4^ Department of Synthetic Biology and Immunology National Institute of Chemistry Ljubljana Slovenia; ^5^ Faculty of Pharmacy University of Ljubljana Ljubljana Slovenia

**Keywords:** basophil migration, CCL2, endothelial monolayer permeability, human mast cells, IL‐33

## Abstract

**Background:**

IL‐33 is involved in allergic processes by promoting the release of various mast cell (MC) chemokines, including CCL2. However, it is yet unclear which specific cell type is primarily responsible for producing CCL2 during acute allergic reactions. This study aims to investigate the role of IL‐33 in promoting CCL2 production in mast cells and assess the effect of MC‐derived CCL2 on basophil migration and endothelial permeability.

**Methods:**

Human blood‐derived MCs (hMCs) were generated from peripheral blood precursors, passively sensitized with IgE, treated with IL‐33, and stimulated with anti‐IgE. The concentrations of nine cytokines known to influence immune cell chemotaxis (CCL2, CCL5, CCL11, MIP‐1α, IL‐8, IL‐10, IL‐13, granulocyte‐macrophage colony‐stimulating factor (GM‐CSF), and vascular endothelial growth factor (VEGF) were assessed in the supernatants of hMCs. Subsequently, we investigated the impact of MC‐derived CCL2 on basophil migration in vitro, as well as its effect on endothelial monolayer permeability using human umbilical vein endothelial cells (HUVECs).

**Results:**

Stimulation with anti‐IgE induced a significant release of CCL2, GM‐CSF, IL‐8 and VEGF from hMCs. Additionally, incubation with IL‐33 overnight increased the production of several cytokines. Mast cell‐derived CCL2 not only enhanced basophil migration in vitro but also increased endothelial monolayer permeability in HUVECs. The effect was reversed by a C–C chemokine receptor type 2 (CCR2) antagonist, indicating the involvement of CCL2 signaling through the CCR2 receptor.

**Conclusions:**

IL‐33 induces the production of chemotactic cytokines in hMCs. Mast cell‐derived CCL2 plays an important role in basophil chemotaxis in vitro and affects endothelial monolayer permeability in the HUVEC model.

## 1 INTRODUCTION

CCL2 was first identified as a chemoattractant for monocytes.^1^ However, it affects several other types of cells.^2^ This chemokine signals to its target cells by binding to and activating the seven transmembrane G‐protein‐coupled receptor C–C chemokine receptor type 2 (CCR2).^3^

CCR2 is expressed on the surface of basophils,^4^ which, alongside mast cells (MCs), are the major effector cells in IgE‐mediated allergic responses.^5^ Both cell types originate from pluripotent CD34^+^ hematopoietic progenitor cells.^6^ While basophils mature in the bone marrow and circulate in the blood, MCs mature in the peripheral tissues where they reside.^6^, ^7^ CCR2 can also be expressed on endothelial cells.^8^ It has been suggested that CCL2 is involved in vascular permeability by inducing specific signaling pathways in adherent junctions of the blood‐brain barrier.^9^, ^10^, ^11^, ^12^ Additionally, CCL2 has been implicated as a vascular permeability factor during lung metastasis.^13^

Activation of MCs and basophils occurs through IgE cross‐linking of cell surface‐expressed FcεRI receptors upon allergen exposure, resulting in the release of preformed mediators,^14^, ^15^ newly synthesized lipids^14^, ^15^, ^16^ and cytokines.^15^, ^17^, ^18^ The latter play a pivotal role in allergic disease pathology by recruiting and activating pro‐inflammatory immune cells.^17^, ^18^, ^19^, ^20^

Upon allergen exposure, epithelial cells produce IL‐33, IL‐25, and thymic stromal lymphopoietin (TSLP), further contributing to the pathogenesis of allergic reactions by enhancing the function of type 2 innate lymphoid cells, basophils, and MCs.^21^, ^22^ IL‐33, in particular, by targeting mainly MCs is implicated in numerous allergic processes.^22^, ^23^

Studies involving experimental allergen challenges in various organs and recent anaphylaxis studies suggest that basophils undergo migration during acute allergic reactions,^24^, ^25^, ^26^, ^27^ although the precise molecular mechanisms underlying this chemotaxis are not yet fully understood. We have previously shown that the serum levels of CCL2 increase during acute allergic reactions, with high levels correlating with CCL2‐mediated basophil chemotaxis and significant migration of circulating basophils.^28^ However, it remains unclear which specific cell type serves as the primary source of CCL2 production during anaphylaxis. In the present study, the role of MCs in CCL2 production is investigated and the effect of CCL2, secreted from human blood‐derived MCs (hMCs), on basophil migration is assessed.

The findings show that CCL2 is a major cytokine produced by hMCs upon anti‐IgE and IL‐33 stimulation, and demonstrate that CCL2 from hMCs not only effectively recruited basophils in vitro but also increased the permeability of endothelial cell monolayers in an in vitro assay. Hence, the findings suggest a dual role for MC‐derived CCL2 in allergic reactions, wherein its production not only influences basophil migration but also affects endothelial cell barrier function by modulating vascular permeability.

## 2 METHODS

### 2.1 Generation of human mast cells from peripheral blood precursors

Healthy donor peripheral blood leukocyte cones were obtained from NHS Blood and Transplant (Manchester, UK) under a material transfer agreement, and used in accordance with a protocol approved by the University of Manchester Research Ethics Committee (UREC ref: 2018‐2696‐5711).

Human mast cells were generated as previously described.^29^ Briefly, peripheral blood mononuclear cells (PBMCs) were isolated from leukocyte cones of healthy blood donors via Ficoll density gradient centrifugation. CD34+/CD117+ precursor cells were purified from PBMCs by using a CD117 positive selection kit (Miltenyi Biotec, Bergisch Gladbach, Germany). Cells were cultured in serum‐free StemSpan II medium (STEMCELL Technologies, Vancouver, BC, Canada) supplemented with 100 U/mL penicillin (Sigma‐Aldrich, St. Louis, MO, USA), 100 μg/mL streptomycin (Sigma‐Aldrich, St. Louis, MO, USA), 50 ng/mL human IL‐6 (Peprotech, Rocky Hill, NJ, USA), 10 ng/mL human IL‐3 (Peprotech, Rocky Hill, NJ, USA) and 100 ng/mL human stem cell factor (STEMCELL Technologies, Vancouver, BC, Canada). After 4 weeks, the cells were transferred to Iscove's Modified Dulbecco's Medium with 2 mM GlutaMAX (Thermo Fisher, Waltham, MA, USA), 50 μmol/L 2‐Mercaptoethanol (Thermo Fisher, Waltham, MA, USA), 0.5% BSA (Thermo Fisher, Waltham, MA, USA), 1% Insulin‐Transferrin‐Selenium (Thermo Fisher, Waltham, MA, USA), 100 U/mL penicillin, 100 μg/mL streptomycin, 50 ng/mL IL‐6 and 100 ng/mL human stem cell factor. Following a culture period of 10 weeks, the mature cell population displayed a proportion exceeding 90% of cells that were identified as CD117+ and FcɛRIa+ cells.

### 2.2 Passive sensitization and stimulation of cultured hMCs

Cultured hMCs (0.5 × 10^6^ cells/mL) were sensitized passively overnight with 1 μg/mL recombinant human IgE myeloma (Merck Life Science UK Limited, Dorset UK, Cat#401152) in the presence or absence of 50 ng/ml IL‐33 (Peprotech, Rocky Hill, NJ, USA, Cat#200‐33). The cells were washed and resuspended in HBSS and either stimulated with 1 μg/mL anti‐IgE (Goat Anti‐Human IgE, LGC SeraCare KPL, Milford, USA, Cat#5210‐0158) or left untreated as control. After incubation for varying periods (1 h or 16 h), the supernatants were collected.

### 2.3 Cytometric bead array (CBA)

CBA Flex Set kit (BD Biosciences, San Jose, CA, USA) was used for the simultaneous detection of nine cytokines (i.e., interleukin [IL‐8, IL‐10, and IL‐13], chemokines (i.e., CCL2, CCL5, CCL11, MIP‐1α), granulocyte‐macrophage colony‐stimulating factor (GM‐CSF) and vascular endothelial growth factor (VEGF) in the supernatants collected after the stimulation of hMCs in the supernatants of the control. The CBA technique was performed according to the instructions provided by the manufacturer. Data were analyzed using the FCAP Array Software (BD Biosciences).

### 2.4 In vitro permeability assay

Endothelial cell permeability was modeled by using HUVEC (Promocell, Heidelberg, Germany) monolayers in a Transwell system. Briefly, HUVEC cells were seeded on collagen‐coated porous polyethylene terephthalate membranes at 200,000 cells/insert and incubated at 37°C in 5% CO2 until a monolayer was formed. Fully confluent monolayers were treated with different vascular permeability factors (recombinant CCL2 [rCCL2], supernatants of hMCs) for 1 h. For blockade of the CCR2 receptor, cells were previously treated with a CCR2 antagonist (Santa Cruz Biotechnology, Dallas, TX, USA). Equal amounts of FITC‐dextran were added to the upper chamber (inserts) in all the experiments and incubated for 20 min. The amount of tracer penetrating through the cell monolayer into the lower chamber was measured using a fluorometer (Synergy, Bio‐Tek, Winooski, VT, USA) at 485 nm. Data were normalized as a percentage of control (cells treated with medium only).

### 2.5 Migration of basophils

We conducted the basophil migration assay using a modified Boyden chamber and polycarbonate membrane cell culture inserts (Corning Inc., New York, NY, USA). Basophils from healthy donors were obtained in the accordance with the approval by The National Medical Ethics Committee of the Republic of Slovenia (No. 0120–189/2019/4), all the donors signed informed consent. Basophils were added to the upper wells, and the supernatants of the hMCs to be tested were placed in the lower wells. The cells were then incubated at 37°C for 150 min. After incubation, we collected the cells that had migrated to the lower wells and quantified them by absolute basophil counts as described before.^28^ Basophil migration was calculated by using the following equation:

Basophil migration (%) = absolute number of migrated basophils/absolute number of seeded basophils × 100.

For control experiments, rCCL2 (Thermo Fisher Scientific, Waltham, MA, USA) was used as a positive control, and a CCR2 antagonist (Santa Cruz Biotechnology, Dallas, TX, USA) was used as a negative control to inhibit CCR2 activation. All experiments were independently performed in triplicate.

## 3 RESULTS

### 3.1 Treatment with IL‐33 increases the production of CCL2, GM‐CSF, IL‐8, and VEGF in hMCs when stimulated with anti‐IgE

To evaluate the effect of IL‐33 on the production of cytokines known to affect immune cell chemotaxis, such as CCL2,^30^ CCL5,^31^ CCL11,^32^ MIP‐1α,^33^ IL‐8,^34^ IL‐10,^35^ IL‐13,^36^ GM‐CSF,^37^ and VEGF,^38^ a set of experiments with IgE‐sensitized hMCs was performed.

To investigate the impact of IL‐33 alone on cytokine production by hMCs, cells were either treated overnight with 50 ng/mL IL‐33 (16 h) or left untreated (control). The concentration of cytokines in the supernatants was measured using the CBA technique.

**FIGURE 1:**
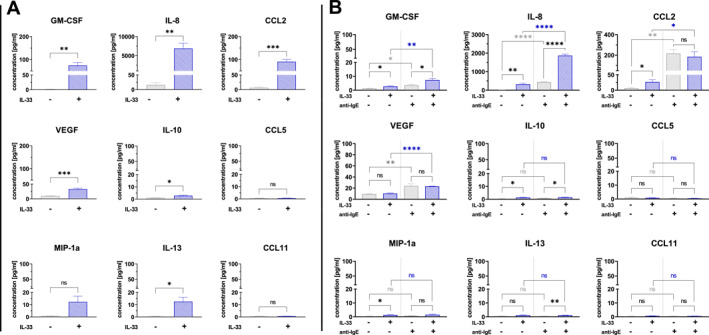
Treatment with IL‐33 increases the production of CCL2, GM‐CSF, IL‐8, and VEGF in hMCs when stimulated with anti‐IgE. (A) IgE‐sensitized hMCs were treated with IL‐33 for 16h (blue) or left untreated in HBSS (gray, control). Production of CCL2, CCL5, CCL11, GM‐CSF, IL‐8, IL‐10, IL‐13, MIP‐1α and VEGF was measured in the collected supernatants. Data are pooled experiment (*n* = 4). Mean ± SEM; 2‐tailed, unpaired *t*‐test. *p < 0.05; **p < 0.01; ***p < 0.001; ****p < 0.0001, (B) IgE‐sensitized hMCs were treated with IL‐33 or left untreated, washed, and then stimulated with 1 μg/mL anti‐IgE or left non‐stimulated in HBSS. Production of CCL2, CCL5, CCL11, GM‐CSF, IL‐8, IL‐10, IL‐13, MIP‐1α and VEGF was measured 1h after stimulation. Data are pooled experiment (*n* = 4). Mean ± SEM; 2‐tailed, unpaired *t*‐test. *p < 0.05; **p < 0.01; ***p < 0.001; ****p < 0.0001.

The results indicated that overnight treatment with IL‐33 caused a significant increase in six out of nine measured concentrations of cytokines produced by hMCs, specifically, CCL2, GM‐CSF, IL‐8, IL‐10, IL‐13, and VEGF (respectively, *n* = 4, *p* = 0.0002; *n* = 4, *p* = 0.0013; *n* = 4, *p* = 0.0023; *n* = 4, *p* = 0.0137; *n* = 4, *p* = 0.0145; *n* = 4, *p* = 0.0004) (Figure 1A).

To investigate whether IL‐33 affects the production of cytokines in stimulated hMCs, we treated hMCs with IL‐33 overnight or left them in medium as a control. Cells were then washed and stimulated with anti‐IgE for 1 h or left unstimulated in the HBSS. The concentration of cytokines in the supernatants was evaluated using the CBA technique.

Concentrations of CCL2 increased significantly in untreated and IgE‐stimulated hMCs supernatants compared to those of untreated and non‐stimulated cells (*n* = 4, *p* = 0.0015). Additionally, a significant increase in the IL‐8 concentrations was observed in supernatants of untreated and anti‐IgE stimulated versus supernatants of untreated and non‐stimulated cells after 1 h (*n* = 4, *p* < 0.0001). Moreover, a somewhat subtle but still significant increase in the concentrations of an increase in GM‐CSF and VEGF concentrations of untreated and anti‐IgE‐stimulated hMCs supernatants was detected (*n* = 4, *p* = 0.0126; *n* = 4, *p* = 0.0093, respectively) (Figure 1B).

In the supernatants of IL‐33 treated and anti‐IgE stimulated hMCs compared to supernatants of IL‐33 treated cells that were left non‐stimulated we observed a significant increase in concentrations of cytokines CCL2, IL‐8, GM‐CSF, and VEGF (respectively, *n* = 4, *p* = 0.0173; *n* = 4, *p* < 0.0001; *n* = 4, *p* = 0.0053; *n* = 4, *p* > 0.0001) (Figure 1B).

IL‐33 treated non‐stimulated hMCs produced significantly higher concentrations of cytokines CCL2, IL‐8, GM‐CSF, IL‐10, and MIP‐1α (*n* = 4, *p* = 0.0302; *n* = 4, *p* = 0.0022; *n* = 4, *p* = 0.0115; *n* = 4, *p* = 0.0211; *n* = 4, *p* = 0.0154, respectively) in comparison to those hMCs that were untreated and non‐stimulated (Figure 1B).

IL‐33 significantly enhanced the production of GM‐CSF, IL‐8, IL‐10, and IL13 (*n* = 4, *p* = 0.0198; *n* = 4, *p* < 0.0001; *n* = 4, *p* = 0.0152; *n* = 4, *p* = 0.0077, respectively) in anti‐IgE stimulated hMCs in comparison to production of cytokines in untreated and anti‐IgE stimulated hMCs (Figure 1B).

Overall, the results suggest that IL‐33 favors hMC cytokine production, previously shown to affect immune cell chemotaxis. These data also demonstrate that IL‐33 treatment in combination with anti‐IgE‐stimulation of hMCs results in an enhanced secretion of various cytokines involved in immune cell chemotaxis.

### 3.2 Basophils show high surface expression of the CCR2 receptor

**FIGURE 2:**
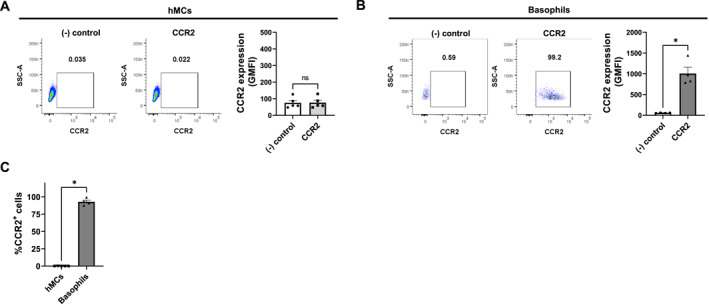
Surface expression of the CCR2 receptor on the surface of major effector cells in IgE‐mediated allergic responses. CCR2 membrane expression was analyzed by flow cytometry on hMCs and basophils. (A) Mast cells showed no expression of CCR2 on their surface (*n* = 5). (B) basophils showed a high surface expression of CCR2 receptor (*n* = 4). Negative control staining ((−) control)) and CCR2 staining (CCR2) representative dot plots are shown as well as the geometric mean fluorescence (GMFI). (C) shows the percentage of CCR2^+^ hMCs and basophils. Data are pooled experiments (hMCs *n* = 5; basophils *n* = 4). Mean ± SEM; 2‐tailed, unpaired *t*‐test; *p < 0.05.

To investigate whether MCs and basophils, major effector cells in IgE‐mediated allergic responses, are targets for CCL2 activities, we used flow cytometric analysis to examine the surface expression of CCR2. Generated hMCs showed no expression of CCR2 on their surface at a steady state (Figures 2A and C). On the other hand, freshly isolated basophils showed a high surface expression of the CCR2 receptor; 92.8% of basophils express CCR2 at their surface (mean, SD: 92.8 ± 4.8%) (Figures 2B and C).

### 3.3 Mast cell‐derived CCL2 enhances basophil migration in vitro

Since hMCs release CCL2 when stimulated and basophils strongly express its receptor, we examined whether MC‐derived CCL2 could prompt basophil chemotaxis. To this purpose, basophils were isolated from healthy donors and a migration assay was performed using a modified Boyden chamber to quantify the basophil count using absolute flow cytometry. Basophils were exposed to supernatants of IL‐33 treated or non‐treated hMCs in the presence or absence of a CCR2 antagonist. rCCL2 was used as a positive control, and Hank's balanced salt solution (HBSS) as a negative control.

**FIGURE 3:**
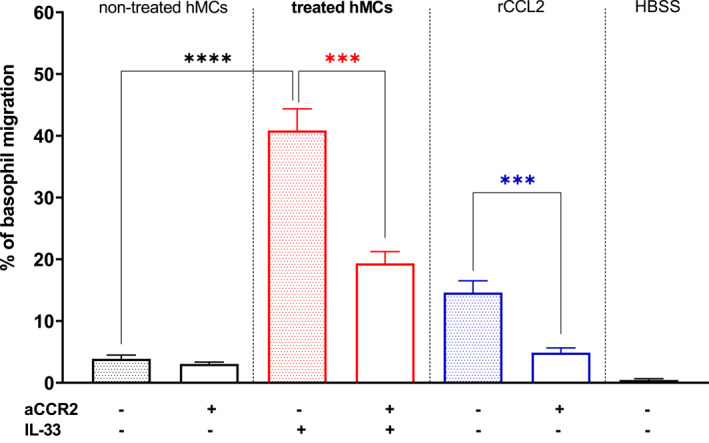
Supernatants of IL‐33 treated hMCs induce CCL2/CCR2‐dependent in vitro basophil migration. After 16h incubation with or without IL‐33 (50 ng/ml), hMCs were washed and left in HBSS for 1h. The supernatants were then collected and used in the migration assay. Basophils were tested for migration toward lower chambers containing supernatant of IL‐33 treated (red) or supernatant of non‐treated hMCs (black) or 10 nM recombinant CCL2 (rCCL2) (positive control, blue). We compared the effect of the supernatant of non‐treated hMCs with the effect of the supernatant of IL‐33 treated hMCs on basophil migration (*p* < 0.0001). The effect of CCL2 in the supernatant of IL‐33 treated hMCs and rCCL2 was blocked by using an antagonist of the CCR2 receptor (aCCR2) (*p* = 0.0001; *p* = 0.0003, respectively). Data are pooled from 4 independent experiments performed in triplicates. Mean ± SEM; 2‐tailed, unpaired *t*‐test.

As shown in Figure 3, we observed a slight increase in basophil migration when exposed to the supernatant of non‐stimulated hMCs compared to the negative control. However, this effect was not reversed upon pretreatment with a CCR2 antagonist (mean, SD: 3.9 ± 2.1% vs. 3.1 ± 1.0%; ns). Conversely, basophil migration increased tenfold in the presence of supernatants of treated hMCs compared with those of non‐treated hMCs (mean, SD: 40.9 ± 10.5% vs. 3.9 ± 2.1%; *p* < 0.0001). Furthermore, this effect was inhibited when cells were pretreated with a CCR2 antagonist (mean, SD: 40.9 ± 10.5% vs. 19.4 ± 5.4%; *p* = 0.0001). Treatment with rCCL2 yielded a similar effect to treatment with supernatant from stimulated hMCs, although the increase in basophil migration was slightly less pronounced (mean, SD: 14.6 ± 5.0%).

These results indicate that some baseline migration might be due to the release of cytokines from hMCs, as the migration of cells treated with only HBSS was almost non‐detectable (mean, SD: 0.5 ± 0.6%). Treatment of hMCs led to higher concentrations of cytokines, which in turn resulted in increased basophil migration in vitro.

The mimicked effect of stimulation with rCCL2 suggests that basophil migration is at least partially attributed to the signaling of CCL2 from hMCs through the CCR2 receptor expressed on basophils (Figure 3).

### 3.4 Mast cell‐derived CCL2 increases endothelial monolayer permeability in HUVECs

To determine whether CCL2 released from IL‐33 treated hMCs could affect the paracellular permeability of the endothelial cell monolayer, HUVECs, known to express CCR2,^39^ were treated with IL‐33 treated hMC supernatant containing CCL2 or with rCCL2 as a positive control. Endothelial permeability was analyzed using a transwell system with an FITC‐labeled dextran tracer.

**FIGURE 4:**
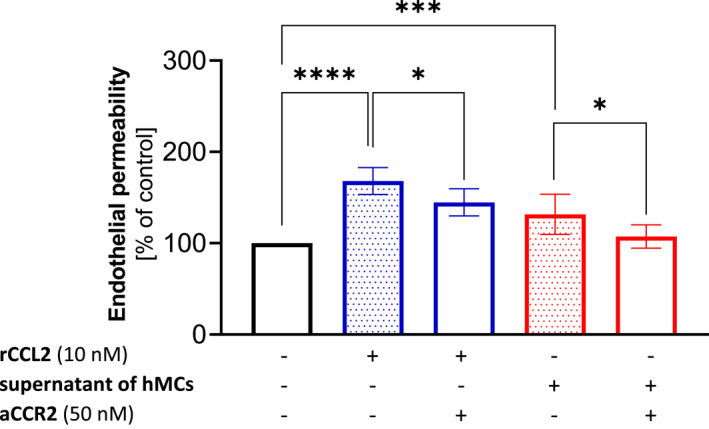
Supernatants of IL‐33 treated hMCs increase endothelial permeability by CCL2/CCR2 dependent mechanism. Endothelial monolayer permeability was measured using a transwell permeability system with a fluorescence‐labeled dextran tracer. HUVECs were treated with supernatants of IL‐33 treated hMCs (red) or rCCL2 (blue) for 60 min. The results of the cells treated with supernatants of treated hMCs were compared with the results of control cells (*n* = 6, ***p = 0.0002). The results of the rCCL2‐treated cells were compared with the results of control cells (*n* = 6, ****p < 0.0001). The effect of CCL2 in the supernatants of IL‐33 treated hMCs and rCCL2 was blocked by using an antagonist of the CCR2 receptor. The results of the blockade were compared with the results of supernatants (*n* = 6, *p = 0.0127) or rCCL2 (*n* = 6, *p = 0.0277). All experiments were independently performed in duplicates. Mean ± SEM; 2‐tailed, unpaired *t*‐test. aCCR2 – CCR2 antagonist, hMCs – human mast cells, rCCL2 – recombinant CCL2.

We observed that the IL‐33 treated hMCs supernatants had an impact on the permeability, as treatment of the HUVEC monolayer with the supernatant for 1 h led to a significant 39% increase in endothelial permeability compared with untreated cells (*n* = 6, *p* = 0.0002). Treatment of the HUVEC monolayer with rCCL2 (10 nM) for 1 h resulted in a significant 68% increase in endothelial permeability compared with untreated cells (*n* = 6, *p* < 0.0001) as shown in Figure 4.

Therefore, it can be concluded that specific molecules in the hMCs supernatant distinctly affect cell permeability. While the permeability effect was less pronounced compared to treatment with rCCL2, this could be due to differences in concentrations of CCL2 as cytokine concentrations in the supernatants were lower than the concentration of rCCL2.

CC chemokines like CCL2 primarily act via the CCR2 receptors, which are members of the family of G protein‐coupled receptors. To determine the involvement of CCR2 receptors in CCL2‐mediated hyper‐permeability, HUVECs were pretreated with CCR2 antagonist for 40 min followed by rCCL2 or hMC supernatants for another hour. Interestingly, the antagonist of CCR2 effectively reduced supernatant‐induced permeability in HUVECs (*n* = 6, *p* = 0.0127), and the same reduction was observed in rCCL2‐induced permeability (*n* = 6, *p* = 0.0277) as shown in Figure 4.

These data demonstrate that CCR2 receptors play an important role in CCL2‐mediated endothelial permeability. Though the permeability was not completely reversed to the level of the control cells, there was a modest, yet significant decrease in monolayer permeability upon treatment with the receptor antagonist, indicating that the effect is at least in part due to signaling through CCR2.

## 4 DISCUSSION

It has been reported that IL‐33 favors MC degranulation and chemokine production amplifying the individual MC response.^40^ Given that IL‐33 is produced by stressed epithelial cells upon allergen exposure, enhancing the MC response may be crucial in allergic processes.^41^ Here, we investigated the effect of IL‐33 on MC‐derived cytokines, previously shown to affect immune cell chemotaxis.

We showed that hMCs secrete several other cytokines besides CCL2 with the ability to affect chemotaxis. Studies showed that high concentrations of IL‐8 were detected in the supernatants of stimulated hMCs. IL‐8, which is mostly known as a neutrophil chemotactic factor,^42^ can also affect basophil migration through CXCR1/CXCR2 receptors, albeit to a lesser extent.^4^

Interestingly, the results in other studies of migration through endothelial monolayer showed that CCL2 induces strong transendothelial migration (TEM); however, no significant TEM was observed in cells stimulated with IL‐8. It is important to note that endothelial cells in the monolayer are firstly HUVEC cells and may not fully recapitulate characteristic of primary endothelial cells in vivo and secondly are capable of autocrine production of various cytokines in response to different stimuli and might disturb the chemotactic gradient and thereby the TEM results.^30^

We showed a more subtle, however also significant, increase in GM‐CSF and VEGF concentrations in stimulated hMC supernatants. GM‐CSF, among other cytokines, was described to have an effect on basophil migration as well as basophil releasability, which may contribute to late‐phase allergic responses.^43^, ^44^ It appears that VEGF not only induces basophil chemotaxis but also plays a role in angiogenesis through the expression of several forms of VEGF and their receptors, creating an autocrine loop.^45^

The observed increase in permeability following treatment with hMC supernatant and rCCL2 confirms the role of CCL2 as a possible mediator of endothelial dysfunction. Although the permeability did not fully revert to the control level, the modest reduction observed suggests that CCR2 signaling is at least partially responsible for the observed effect. Nevertheless, the results of our study confirm the findings of previous research that CCL2 is involved in vascular permeability.^13^ Our study has some limitations; for example, we focused only on the inhibition of the CCL2 effect on the permeability, and we only performed it on the HUVEC model. Therefore, it is important to notice that in other study IL‐8 regulated the permeability of the endothelium by down‐regulating tight junction components in human vascular endothelial cell lines, which are hybridoma cell lines between HUVECs and the epithelioma A549 cells.^46^

The effect of CCL2/CCR2 signaling on permeability may not be limited to allergic response reactions, as several other studies underline this specific effect in a different context. It has been reported that CCL2, through CCR2 present on endothelial cells, induces brain endothelial hyperpermeability via Rho/PKCα signal pathway interactions.^9^, ^10^ CCL2‐mediated disruption of VE‐cadherin, CCL2‐induced signaling between PECAM‐1 and the adherens junctions, and CCL2‐associated increases in PECAM‐1 expression and cell surface localization were demonstrated in human cerebral endothelial cells.^11^ Furthermore, CCL2 stimulation of pulmonary endothelial cells induced endothelial cell retraction and vascular leakiness that was blocked by the addition of a CCR2 inhibitor.^13^

The results presented in this study have broader implications for various pathological conditions characterized by vascular dysfunction. Increased vascular permeability is a hallmark of inflammation and plays a crucial role in the pathogenesis of diseases such as anaphylaxis, atherosclerosis, asthma, and cancer.^47^, ^48^ Some reports show successful implications of therapeutic interventions targeting the CCL2/CCR2 axis. Inhibition or blockade of the CCL2/CCR2 signaling axis has thus been an area of interest for cancer therapy. In murine tumor and metastasis models CCR2 antagonism in combination with anti‐PD‐1 therapy leads to sensitization and enhanced tumor response over anti‐PD‐1 monotherapy.^49^ Knockout or blockade of CCL2/CCR2 inhibits primary liver tumor and metastatic growth leading to prolonged survival.^50^ More interestingly, blockade of CCL2/CCR2 signaling provides protective immunity in murine models of OVA‐induced allergic asthma.^51^

In conclusion, IL‐33 favors mast cell degranulation and increases the production of several cytokines, for example, CCL2, IL‐8, VEGF and GM‐CSF. Mast cell‐derived CCL2‐mediated basophil chemotactic activity suggests an important role of CCL2 in basophil recruitment. The parallel effect on endothelial permeability in the HUVEC model suggests that a disrupted endothelial barrier might allow basophils to migrate freely to the site of inflammation where effector functions may be subsequently executed. Further investigation of the downstream signaling pathways activated by CCL2‐CCR2 interactions on several other models could provide a more comprehensive understanding of the mechanisms underlying endothelial barrier disruption.

## AUTHOR CONTRIBUTIONS

**Maruša Rihar:** Conceptualization; methodology; validation; data curation; formal analysis; investigation; writing—original draft; visualization. **Rajia Bahri:** Conceptualization; methodology; formal analysis; writing—review and editing. **Vida Forstnerič:** Conceptualization; methodology; writing—review and editing. **Silvia Bulfone‐Paus:** Conceptualization; supervision; resources; funding acquisition; writing—review and editing. **Peter Korošec:** Conceptualization; methodology; writing—review and editing; resources; supervision; project administration; funding acquisition; supervision. All authors contributed to manuscript revision and read and approved the submitted version.

## CONFLICT OF INTEREST STATEMENT

The authors declare no conflicts of interest.

## Data Availability

Data will be made available on request.

## References

[1] Matsushima K , Larsen CG , DuBois GC , Oppenheim JJ . Purification and characterization of a novel monocyte chemotactic and activating factor produced by a human myelomonocytic cell line. J Exp Med. 1989;169(4):1485‐1490. 10.1084/jem.169.4.1485 2926331 PMC2189236

[2] Gschwandtner M , Derler R , Midwood KS . More than just attractive: how CCL2 influences myeloid cell behavior beyond chemotaxis. Front Immunol. 2019;10. 10.3389/fimmu.2019.02759 PMC6923224 31921102

[3] Charo IF , Myers SJ , Herman A , Franci C , Connolly AJ , Coughlin SR . Molecular cloning and functional expression of two monocyte chemoattractant protein 1 receptors reveals alternative splicing of the carboxyl‐terminal tails. Proc Natl Acad Sci USA. 1994;91(7):2752‐2756. 10.1073/pnas.91.7.2752 8146186 PMC43448

[4] Iikura M , Miyamasu M , Yamaguchi M , et al. Chemokine receptors in human basophils: inducible expression of functional CXCR4. J Leukoc Biol. 2001;70(1):113‐120. http://www.ncbi.nlm.nih.gov/pubmed/11435493 11435493

[5] Costa JJ . The cells of the allergic response. JAMA. 1997;278(22):1815. 10.1001/jama.1997.03550220021005 9396642

[6] Kirshenbaum AS , Goff JP , Kessler SW , Mican JM , Zsebo KM , Metcalfe DD . Effect of IL‐3 and stem cell factor on the appearance of human basophils and mast cells from CD34+ pluripotent progenitor cells. J Immunol. 1992;148(3):772‐777. 10.4049/jimmunol.148.3.772 1370517

[7] Kirshenbaum AS , Kessler SW , Goff JP , Metcalfe DD . Demonstration of the origin of human mast cells from CD34+ bone marrow progenitor cells. J Immunol. 1991;146(5):1410‐1415. http://www.ncbi.nlm.nih.gov/pubmed/1704394 1704394

[8] Weber KSC , Nelson PJ , Gro Hjoseph , Weber C . Implications for MCP‐1 mediated wound injury repair and in vivo. Arterioscler Thromb. 1999:2085‐2093. Published online. 10.1161/01.atv.19.9.2085 10479649

[9] Stamatovic SM , Keep RF , Kunkel SL , Andjelkovic AV . Potential role of MCP‐1 in endothelial cell tight junction `opening’: Signaling Via Rho and Rho Kinase. J Cell Sci. 2003;116(22):4615‐4628. 10.1242/jcs.00755 14576355

[10] Stamatovic SM , Dimitrijevic OB , Keep RF , Andjelkovic AV . Protein kinase Cα‐RhoA cross‐talk in CCL2‐induced alterations in brain endothelial permeability. J Biol Chem. 2006;281(13):8379‐8388. 10.1074/jbc.M513122200 16439355

[11] Roberts TK , Eugenin EA , Lopez L , et al. CCL2 disrupts the adherens junction: Implications for Neuroinflammation. Lab Invest. 2012;92(8):1213‐1233. 10.1038/labinvest.2012.80 22641100 PMC3409314

[12] Guo F , Xu D , Lin Y , et al. Chemokine CCL2 contributes to BBB disruption via the p38 MAPK signaling pathway following acute intracerebral hemorrhage. FASEB (Fed Am Soc Exp Biol) J. 2020;34(1):1872‐1884. 10.1096/fj.201902203RR 31914700

[13] Roblek M , Protsyuk D , Becker PF , et al. CCL2 is a vascular permeability factor inducing CCR2‐dependent endothelial retraction during lung metastasis. Mol Cancer Res. 2019;17(3):783‐793. 10.1158/1541-7786.MCR-18-0530 30552233 PMC6445360

[14] Stone KD , Prussin C , Metcalfe DD . IgE, mast cells, basophils, and eosinophils. J Allergy Clin Immunol. 2010;125(2):S73‐S80. 10.1016/j.jaci.2009.11.017 20176269 PMC2847274

[15] Varricchi G , Raap U , Rivellese F , Marone G , Gibbs BF . Human mast cells and basophils—how are they similar how are they different? Immunol Rev. 2018;282(1):8‐34. 10.1111/imr.12627 29431214

[16] Nakamura T . The roles of lipid mediators in type I hypersensitivity. J Pharmacol Sci. 2021;147(1):126‐131. 10.1016/j.jphs.2021.06.001 34294363

[17] Gordon JR , Burd PR , Galli SJ . Mast cells as a source of multifunctional cytokines. Immunol Today. 1990;11:458‐464. 10.1016/0167-5699(90)90176-A 2073318

[18] Galli SJ , Costa JJ . Mast‐cell—leukocyte cytokine cascades in allergic inflammation. Allergy. 1995;50(11):851‐862. 10.1111/j.1398-9995.1995.tb02490.x 8748716

[19] Borish LC , Steinke JW . 2. Cytokines and chemokines. J Allergy Clin Immunol. 2003;111(2 (Suppl 2)):S460‐S475. 10.1067/mai.2003.108 12592293

[20] Yamaguchi M , Koketsu R , Suzukawa M , Kawakami A , Iikura M . Human basophils and cytokines/chemokines. Allergol Int. 2009;58(1):1‐10. 10.2332/allergolint.08-RAI-0056 19153531

[21] Ohno T , Morita H , Arae K , Matsumoto K , Nakae S . Interleukin‐33 in allergy. Allergy: European Journal of Allergy and Clinical Immunology. 2012;67(10):1203‐1214. 10.1111/all.12004 22913600

[22] Chu DK , Llop‐Guevara A , Walker TD , et al. IL‐33, but not thymic stromal lymphopoietin or IL‐25, is central to mite and peanut allergic sensitization. J Allergy Clin Immunol. 2013;131(1):187‐200.e8. 10.1016/j.jaci.2012.08.002 23006545

[23] Chan BCL , Lam CWK , Tam LS , Wong CK . IL33: roles in allergic inflammation and therapeutic perspectives. Front Immunol. 2019;10(MAR). 10.3389/fimmu.2019.00364 PMC6409346 30886621

[24] Irani AMA , Huang C , Xia HZ , et al. Immunohistochemical detection of human basophils in late‐phase skin reactions. J Allergy Clin Immunol. 1998;101(3):354‐362. 10.1016/s0091-6749(98)70248-9 9525452

[25] Nouri‐Aria KT , Jacobson MR , O’Brien F , et al. Basophil recruitment and IL‐4 production during human allergen‐induced late asthma. J Allergy Clin Immunol. 2001;108(2):205‐211. 10.1067/mai.2001.117175 11496235

[26] Korosec P , Turner PJ , Silar M , et al. Basophils, high‐affinity IgE receptors, and CCL2 in human anaphylaxis. J Allergy Clin Immunol. 2017;140(3):750‐758.e15. 10.1016/j.jaci.2016.12.989 28342911 PMC5587023

[27] Korošec P , Gibbs BF , Rijavec M , Custovic A , Turner PJ . Important and specific role for basophils in acute allergic reactions. Clin Exp Allergy. 2018;48(5):502‐512. 10.1111/cea.13117 29431885 PMC5947573

[28] Vantur R , Rihar M , Koren A , et al. Chemokines during anaphylaxis: The Importance of CCL2 and CCL2‐Dependent Chemotactic Activity for Basophils. Clin Transl Allergy. 2020;10(1):63. 10.1186/s13601-020-00367-2 33317619 PMC7737350

[29] Bahri R , Custovic A , Korosec P , et al. Mast cell activation test in the diagnosis of allergic disease and anaphylaxis. J Allergy Clin Immunol. 2018;142(2):485‐496.e16. 10.1016/j.jaci.2018.01.043 29518421 PMC6075471

[30] Iikura M , Ebisawa M , Yamaguchi M , et al. Transendothelial migration of human basophils. J Immunol. 2004;173(8):5189‐5195. 10.4049/jimmunol.173.8.5189 15470064

[31] Chan O , Burke JD , Gao DF , Fish EN . The chemokine CCL5 regulates glucose uptake and AMP kinase signaling in activated T cells to facilitate chemotaxis. J Biol Chem. 2012;287(35):29406‐29416. 10.1074/jbc.M112.348946 22782897 PMC3436201

[32] Rothenberg ME , Ownbey R , Mehlhop PD , et al. Eotaxin triggers eosinophil‐selective chemotaxis and calcium flux via a distinct receptor and induces pulmonary eosinophilia in the presence of interleukin 5 in mice. Mol Med. 1996;2(3):334‐348 8784786 PMC2230145

[33] Schall TJ , Bacon K , Camp RDR , Kaspari JW , Goeddel DV . Human macrophage inflammatory protein Cr (MIP‐Lc∼) and MIP‐I∼ chemoklnes attract distinct populations of lymphocytes. J Exp Med. 1993;177(6):1821‐1826. http://rupress.org/jem/article‐pdf/177/6/1821/1103957/1821.pdf 7684437 10.1084/jem.177.6.1821 PMC2191042

[34] Henkels KM , Frondorf K , Gonzalez‐Mejia ME , Doseff AL , Gomez‐Cambronero J . IL‐8‐induced neutrophil chemotaxis is mediated by Janus kinase 3 (JAK3). FEBS Lett. 2011;585(1):159‐166. 10.1016/j.febslet.2010.11.031 21095188 PMC3021320

[35] Ogawa Y , Duru E , Ameredes B . Role of IL‐10 in the resolution of airway inflammation. Curr Mol Med. 2008;8(5):437‐445. 10.2174/156652408785160907 18691071 PMC9159958

[36] Doran E , Cai F , Holweg CTJ , Wong K , Brumm J , Arron JR . Interleukin‐13 in asthma and other eosinophilic disorders. Front Med. 2017;4(SEP). 10.3389/fmed.2017.00139 PMC5627038 29034234

[37] Castellani S , D’Oria S , Diana A , et al. G‐CSF and GM‐CSF modify neutrophil functions at concentrations found in cystic fibrosis. Sci Rep. 2019;9(1):12937. 10.1038/s41598-019-49419-z 31506515 PMC6736848

[38] Shin JY , Yoon IH , Kim JS , Kim B , Park CG . Vascular endothelial growth factor‐induced chemotaxis and IL‐10 from T cells. Cell Immunol. 2009;256(1‐2):72‐78. 10.1016/j.cellimm.2009.01.006 19249018

[39] Salcedo R , Ponce ML , Young HA , et al. Human endothelial cells express CCR2 and respond to MCP‐1: direct role of MCP‐1 in angiogenesis and tumor progression. Blood. 2000;96(1):34‐40. 10.1182/blood.v96.1.34 10891427

[40] Joulia R , L’Faqihi FE , Valitutti S , Espinosa E . IL‐33 fine tunes mast cell degranulation and chemokine production at the single‐cell level. J Allergy Clin Immunol. 2017;140(2):497‐509.e10. 10.1016/j.jaci.2016.09.049 27876627

[41] Hammad H , Lambrecht BN . Barrier epithelial cells and the control of type 2 immunity. Immunity. 2015;43(1):29‐40. 10.1016/j.immuni.2015.07.007 26200011

[42] Hammond ME , Lapointe GR , Feucht PH , et al. IL‐8 induces neutrophil chemotaxis predominantly via type I IL‐8 receptors. J Immunol. 1995;155(3):1428‐1433. 10.4049/jimmunol.155.3.1428 7636208

[43] Tanimoto Y , Takahashi K , Kimura I . Effects of cytokines on human basophil chemotaxis. Clin Exp Allergy. 1992;22(11):1020‐1025. 10.1111/j.1365-2222.1992.tb03031.x 1334781

[44] Yoshimura‐Uchiyama C , Yamaguchi M , Nagase H , et al. Comparative effects of basophil‐directed growth factors. Biochem Biophys Res Commun. 2003;302(2):201‐206. 10.1016/S0006-291X(03)00153-0 12604332

[45] de Paulis A , Prevete N , Fiorentino I , et al. Expression and functions of the vascular endothelial growth factors and their receptors in human basophils. J Immunol. 2006;177(10):7322‐7331. 10.4049/jimmunol.177.10.7322 17082651

[46] Yu H , Huang X , Ma Y , et al. Interleukin‐8 regulates endothelial permeability by down‐regulation of tight junction but not dependent on integrins induced focal adhesions. Int J Biol Sci. 2013;9(9):966‐979. 10.7150/ijbs.6996 24155670 PMC3805902

[47] Carraway RE , Cochrane DE . Enhanced vascular permeability is hypothesized to promote inflammation‐induced carcinogenesis and tumor development via extravasation of large molecular proteins into the tissue. Med Hypotheses. 2012;78(6):738‐743. 10.1016/j.mehy.2012.02.021 22459481

[48] Claesson‐Welsh L . Vascular permeability—the essentials. Upsala J Med Sci. 2015;120(3):135‐143. 10.3109/03009734.2015.1064501 26220421 PMC4526869

[49] Tu MM , Abdel‐Hafiz HA , Jones RT , et al. Inhibition of the CCL2 receptor, CCR2, enhances tumor response to immune checkpoint therapy. Commun Biol. 2020;3(1):720. 10.1038/s42003-020-01441-y 33247183 PMC7699641

[50] Li X , Yao W , Yuan Y , et al. Targeting of tumour‐infiltrating macrophages via CCL2/CCR2 signalling as a therapeutic strategy against hepatocellular carcinoma. Gut. 2017;66(1):157‐167. 10.1136/gutjnl-2015-310514 26452628

[51] Jiang S , Wang Q , Wang Y , Song X , Zhang Y . Blockade of CCL2/CCR2 signaling pathway prevents inflammatory monocyte recruitment and attenuates OVA‐Induced allergic asthma in mice. Immunol Lett. 2019;214:30‐36. 10.1016/j.imlet.2019.08.006 31454522

